# Decreased in n‐3 DHA enriched triacylglycerol in small extracellular vesicles of diabetic patients with cardiac dysfunction

**DOI:** 10.1111/1753-0407.13457

**Published:** 2023-08-18

**Authors:** Wei Ding, Xuejuan Zhang, Dandan Xiao, Wenguang Chang

**Affiliations:** ^1^ Department of General Medicine, The Affiliated Hospital, College of Medicine Qingdao University Qingdao China; ^2^ School of Basic Medical Sciences, College of Medicine Qingdao University Qingdao China; ^3^ Institute for Translational Medicine, The Affiliated Hospital, College of Medicine Qingdao University Qingdao China

**Keywords:** cardiac dysfunction, DHA, extracellular vesicles, insulin resistance, triacylglycerol, 心脏功能障碍, DHA, 细胞外囊泡, 胰岛素抵抗, 三酰甘油

## Abstract

**Purpose:**

Diabetic cardiomyopathy is the leading cause of death in diabetic patients, and the mechanism by which factors other than hyperglycemia contribute to the development of diabetic cardiomyopathy is unknown. Serum small extracellular vesicles (sEVs) carry bioactive proteins or nuclei, which enter into remote tissues and modulate cell functions. However, in diabetic conditions, the changes of lipids carried by sEVs has not been identified. Our study aims to explore the changes of lipids in sEVs in diabetic patients with cardiovascular disease, we hope to provide new ideas for understanding the role of lipid metabolism in the pathogenesis of diabetic cardiomyopathy.

**Methods:**

SEVs samples derived from serum of health controls (Ctrl), diabetic patients without cardiovascular diseases (DM), and diabetic patients with cardiovascular diseases (DM‐CAD) were used for lipidomics analysis. Because AC16 cells are also treated with those sEVs to confirm the entrance of cells and effects on insulin sensitivity, a lipidomics analysis on cells was also performed.

**Results and Conclusions:**

In this study, we found that docosahexaenoic acid (DHA)‐triacylglycerides of sEVs from serums of DM‐CAD patients decreased significantly, and those sEVs could enter into AC16 cells and diminish insulin sensitivity. In addition, DHA‐triacylglycerides were also decreased in cells treated with sEVs from DM‐CAD. Therefore, DHA‐triacylglycerides carried by sEVs may mediate intercellular signaling and be associated with the incidence of diabetic cardiovascular complications.

## Graphical abstract

**Highlights**

1. Lipid profiles of small extracellular vesicles (sEVs) in patients with diabetes or diabetic cardiovascular disease are rarely reported.
2. n‐3 docosahexaenoic acid (DHA)‐enriched triacylglycerol (TG) was found to decrease in sEVs from serum of patients with diabetic cardiovascular disease (DM‐CAD).
3. DM‐CAD sEVs diminished activation of AKT in cultured cardiac cells, and showed the same decrease in n‐3 DHA‐TG, suggesting n‐3 DHA‐TG may be associated with diabetic cardiac dysfunction.

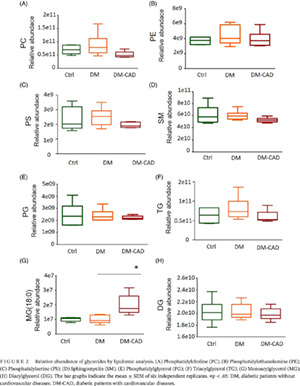

## 摘要

背景:糖尿病心肌病是糖尿病患者死亡的主要原因，除高血糖外，其他因素导致糖尿病心肌病发展的机制尚不清楚。血清sEVs携带生物活性蛋白质或细胞核，进入远端组织并调节细胞功能。然而，在糖尿病条件下，sEVs携带的脂质变化尚未确定。我们的研究旨在探索心血管疾病糖尿病患者sEVs中脂质的变化，我们希望为理解脂质代谢在糖尿病心肌病发病机制中的作用提供新的思路

方法:对健康对照(Ctrl)、无心血管疾病的糖尿病患者(DM)和患有心血管疾病的糖尿病患者(DM‐CAD)血清来源的SEVs样本进行脂质组学分析。以这些sEVs处理AC16细胞以确认其进入细胞和对胰岛素敏感性的影响，对细胞进行脂质组学分析

结果:本研究发现，DM‐CAD患者血清中sEVs的DHA‐三酰甘油显著降低，这些sEVs可以进入AC16细胞并降低胰岛素敏感性。此外，用DM‐CAD患者血清中sEVs处理的细胞中DHA‐三酰甘油也降低

结论:sEVs携带的DHA‐三酰甘油可能介导细胞间信号传导，并与糖尿病心血管并发症的发生有关

## 1 INTRODUCTION

The number of patients with type 2 diabetes (T2D) is estimated to reach 780 million by 2045.^1^ Cardiovascular complication is the main reason for death in T2D patients. Unfortunately, underdiagnosis causes most T2D patients to develop irreversible heart failure by the time symptoms emerge.^2^, ^3^, ^4^ Thus, it is important to explore the mechanisms that mediate the development of cardiovascular disease in diabetic patients. Dysregulation of glucose metabolism and lipid metabolism is the main pathological manifestation of T2D. Excess glucose and lipid are proven to diminish normal endothelial function, destruct pancreatic cells, and induce insulin resistance.^5^, ^6^ In clinical practice, hypoglycemic agents and lipid‐lowering agents are first‐line drugs for the treatment of diabetes.^7^ However, even in patients with good blood sugar control, the incidence rate of cardiovascular complications is still high, indicating more sophisticated regulation metabolism is involved in the progress of diabetic cardiomyopathy.^8^

Small extracellular vesicles (sEVs) are double‐membrane vesicles that are released into extracellular spaces by almost all types of cells. They cannot replicate and do not have functional nuclei. sEVs are defined by size as 50–200 nm, and they are mostly produced by the fusion of multivesicular body (MVB) with the plasma membrane, which is also known as exosomes. However, some EVs are produced by direct budding from the plasma membrane, which also has size of 50–200 nm, and they cannot be distinguished by current isolation methods,^9^ so the term of sEVs represents vesicles (50–200 nm) that are generated by both pathways. In recent years, many studies have shown that sEVs carry bioactive cargoes, including lipids, proteins, and nucleic acids, which mediate cell–cell communication and regulate biological functions in recipient cells.^10^, ^11^ In diabetic condition, serum sEVs were shown to carry sonic hedgehog, a protein that modulates immunity, or arginase1 to induce endothelial dysfunction.^12^ In addition, numbers of noncoding RNA are shown to carry by sEVs and are associated with incidence of diabetic complications.^13^

The changes in lipid profiles of sEVs are proven to be associated with the progress of diseases. For example, levels of phosphatidylcholine (PC), phosphatidylethanolamine (PE), phosphatidylglycerol (PG), and lyso‐PC of plasma sEVs from psoriatic patients were found to be increased compared to healthy control.^14^ Unsaturated fatty acid‐enriched lyso‐PC concentration of sEVs derived from intestinal epithelial cells increased after hypoxia and reoxygenation injury.^15^ Additionally, sEVs from the nonmetastatic cell line displayed a marked increase in PC (34:1), PE (36:2), sphingomyelin (SM; d18:1/16:0), hexosylceramide (HexCer; d18:1/24:0), and HexCer (d18:1/24:1) compared with controls, whereas these same lipids species were decreased in the metastatic cell line.^16^ Therefore, the determination of lipid alterations of plasma sEVs is useful for the diagnosis. However, lipid profiles of sEVs in patients with diabetes or diabetic cardiovascular disease are rarely reported. In our experiment, we found that some species of triglycerides in serum sEVs of diabetes patients changed significantly with the lipidomics method, and we confirmed the changes in cell experiments. These results could provide new ideas for the early diagnosis of diabetes cardiovascular complications.

## 2 MATERIALS AND METHODS

### 2.1 Serum sample preparation for lipidomic analysis

We collected 20 blood samples from each of three groups: health controls (Ctrl), diabetic patients without cardiovascular diseases (DM), and diabetic patients with cardiovascular diseases (DM‐CAD). Then we selected six cases from each group of these samples for subsequent lipidomics analysis. The selection criteria included (1) all groups were of the same age (56–62 years); (2) all groups had no hepatitis, cancer, or genetic diseases; (3) DM group patients had no complications of kidney disease or eye disease; and (4) the cardiovascular disorders in DM‐CAD group patients are mainly characterized by decreased diastolic function. The body mass index (BMI) values and antidiabetic prescriptions that were used for those patients are summarized in Table 1.

**TABLE 1:** BMI and prescriptions used in the samples for lipidomic analysis.

Groups	Healthy control (*n* = 6)	Diabetic without cardiovascular diseases DM (*n* = 6)	Diabetic with cardiovascular diseases DM‐CAD (*n* = 6)
BMI	24.4 ± 1.35	24.1 ± 1.48	25.9 ± 2.03
Oral antidiabetic prescriptions	None	Two or three oral anti‐diabetic drugs in combination: (1) Dapagliflozin, 10 mg qd; (2) Saxagliptin, 5 mg qd; (3) Metformin, 500 mg bid; (4) Glimepirid, 4 mg qd; (5) Acarbose 50 mg tid;	Two or three oral anti‐diabetic drugs in combination: (1) Dapagliflozin, 10 mg qd; (2) Saxagliptin, 5 mg qd; (3) Metformin, 500 mg bid (4) Glimepirid, 4 mg qd; (5) Acarbose 50 mg tid;
Insulin	None	None	None

Abbreviations: BMI, body mass index; DM‐CAD, diabetes patients with cardiovascular disease.

### 2.2 Isolation and analysis of sEVs

Plasma‐derived sEVs were isolated using the Exoeasy Maxi kit (Qiagen, Cat#76064) according to the manufacturer's protocol. The protein concentration of sEVs was measured using a Qubit Protein Assay kit (Molecular Probes from Thermo Fisher Scientific). The particle number of sEVs was measured by NanoSight LM10 with NTA2.3 Analytical software (NanoSight, Wiltshire, UK).

For electron microscopy, the isolated sEVs were dropped onto the carbon support membrane copper mesh and let stand for 3–5 min, then 2% phosphotungstic acid was dropped onto the carbon support membrane copper mesh for 2–3 min, then observed under a transmission electron microscope and images collected for analysis by transmission electron microscope (HITACHI, HT7700).

### 2.3 Cell culture

Human cardiac cell line AC16 was obtained from Cell Resource Center (IBMS, Beijing). Cells were maintained in high glucose DMEM with 10% FBS, 2% l‐glutamine, 10% sodium bicarbonate, 10% sodium pyruvate, 5% HEPES, 1% penicillin/streptomycin, and 1% gentamycin in an incubator (37°C, 5% CO_2_).

### 2.4 Lipid extraction

To samples of EVs 100 μL each, 200 μL ice 75% methanol was added. The samples were placed in an ice bath ultrasound for 15 min, 1 mL of ice MTBE was added for sufficient vortex oscillation, then they were rotated and mixed in a 4°C refrigerator for 1 h. After continuing ice bath ultrasound for 15 min, 200 μL water were added into the tube, standing at room temperature for 10 min, centrifuged at 4°C 14 000 g for 15 min. The layered upper liquid was removed, then the samples were dried with nitrogen gas.

### 2.5 UHPLC‐MS/MS (QE plus) based nontargeted lipidomics analysis

The sample was placed in an automatic sampler and the SHIMADZU‐LC30 ultra‐high performance liquid chromatography system (UHPLC) was used, using ACQUITY UPLC‐HSS C18 (2.1 × 150 mm, 2.5 μm) (Waters, Milford, MA, USA) chromatographic column. The injection volume was 5 μL, column temperature 40°C, flow rate 0.3 mL/min; the mobile phase consisted of (a) 0.77 g ammonium formate, ammonium formate, acetonitrile, and water (acetonitrile: water = 6:4, v/v); (b) acetonitrile and isopropanol solution (acetonitrile: isopropanol = 1:9, v/v). The gradient elution procedure was as follows: 0–2 min, B changes linearly from 30% to 32%; 2–6 min, B changes linearly from 32% to 45%; 6–8 min, B linearly changes from 45% to 52%; 8–12 min, B changes linearly from 52% to 58%; 12–15 min, B changes linearly from 58% to 66%; 15–18 min, B changes linearly from 66% to 70%; 18–21 min, B linearly changes from 70% to 97%; 21–25 min, B maintained at 97%; 25–26 min, B linearly changes from 97% to 32%; 26–30 min, B maintained at 32%. During the entire analysis process, the sample was placed in a 6°C automatic sampler. To avoid the impact caused by signal fluctuations in the instrument's detection, a random sequence is used for continuous sample analysis using the influence caused by follow‐up. Sequential analysis of samples is carried out on every other machine in the queue.

For mass spectrometry conditions: electron eneneba spray ionization positive and negative modes were used for detection respectively. The sample was tested in both positive and negative modes. After separation by UHPLC, the sample was analyzed using a Q Active plus mass spectrometer (Thermo Scientific™) perform mass spectrometry analysis. Positive mode: Heater Temperature 300°C, Sheath Gas Flow rate, 45arb, Aux Gas Flow rate, 15 arb, Sweep Gas Flow rate, 1 arb, spray voltage, 3.0 kv, Capillary Temperature, 350°C, S‐Lens RF Level, 50% Scan ranges: 200–1500; Negative mode: Heater Temperature 300°C, Sheath Gas Flow rate, 45 arb, Aux Gas Flow rate, 15 arb, Sweep Gas Flow rate, 1arb, spray voltage, 3.5kv, Capillary Temperature, 350°C, S‐Lens RF Level, 50% Scan ranges:200–1500.

### 2.6 Supplementation of sEVs to AC16 cells

AC16 cells were seeded at 1 × 10^4^ cells/mL/well in 6‐well culture slides (BD Falcon, Franklin Lakes, NJ, USA) for the uptake assay, 10 μg of PKH67‐stained sEVs based on protein amount were supplemented to AC16 and were incubated for 6 h. Cells were fixed with 4% paraformaldehyde phosphate buffer solution (Wako, Osaka, Japan) for 10 min and mounted with Vecta Shield mounting medium with DAPI (Vector Laboratories, Burlingame, CA, USA). sEV uptake was observed by confocal fluorescence microscopy (FLUOVIEW FV10i, Olympus, Tokyo, Japan).

For insulin stimulation assays, AC16 was supplemented with 10 μg protein of unstained control‐sEVs or DM‐CAD‐sEVs and incubated for 24 h. After washing with PBS, cells were stimulated by 100 nM insulin (M9194, AbMole, USA) for 15 mins, then lysed by Mammalian Protein Extraction Reagent (Pierce from Thermo Fisher Scientific). The supernatants after centrifugation at 14 000 *g*, 5 min of cell lysates were used for protein assays.

### 2.7 Determination of protein expression

Quantitative analysis of AKT, p‐AKT(Ser473), CD63, and TSG101 expression was performed as previously described. p‐AKT(Ser473) (Cell Signaling Technology) 1:1000; p‐AKT(Thr308) (affinity‐AF3262) 1:1000; AKT(Cell Signaling Technology) 1:1000; β‐actin (Santa Cruz, CA, USA) 1:2000; and CD9 (Abcam, ab92726) 1:1000 and TSG101(Abcam, ab83) 1:1000 were used as primary antibodies. The secondary antibodies were obtained from Santa Cruz Biotechnology (Santa Cruz, CA, USA). Protein was visualized with enhanced chemiluminescence solution, and images were generated with a Bio‐Rad Imaging system.

### 2.8 Assay of 2‐deoxy‐2‐((7‐nitro‐2,1,3‐benzoxadiazole‐4‐yl) amino)‐d‐glucose (2‐NBDG) glucose uptake

After cell were treated with sEVs as indicated for 24 h, 2‐NBD‐glucose (M6327, AbMole, USA) 200 μmol/L in PBS was added with or without insulin at 100 nmol/L, and the cells were then incubated for an additional 30 min. Glucose uptake was stopped with three washes with ice‐cold PBS. The fluorescence intensity of the cells was recorded at 488/520 nm by a fluorescence microplate reader.

### 2.9 Statistical analysis

Software MSDAIL (Version 4.0.9) was used for lipid identification and quantification processing. One‐way analysis of variance was used for multiple comparisons. A value of *p* < .05 was considered indicative of significance.

## 3 RESULTS

### 3.1 Characterization of sEVs isolated from the serum of T2D patients with cardiovascular disease

**FIGURE 1:**
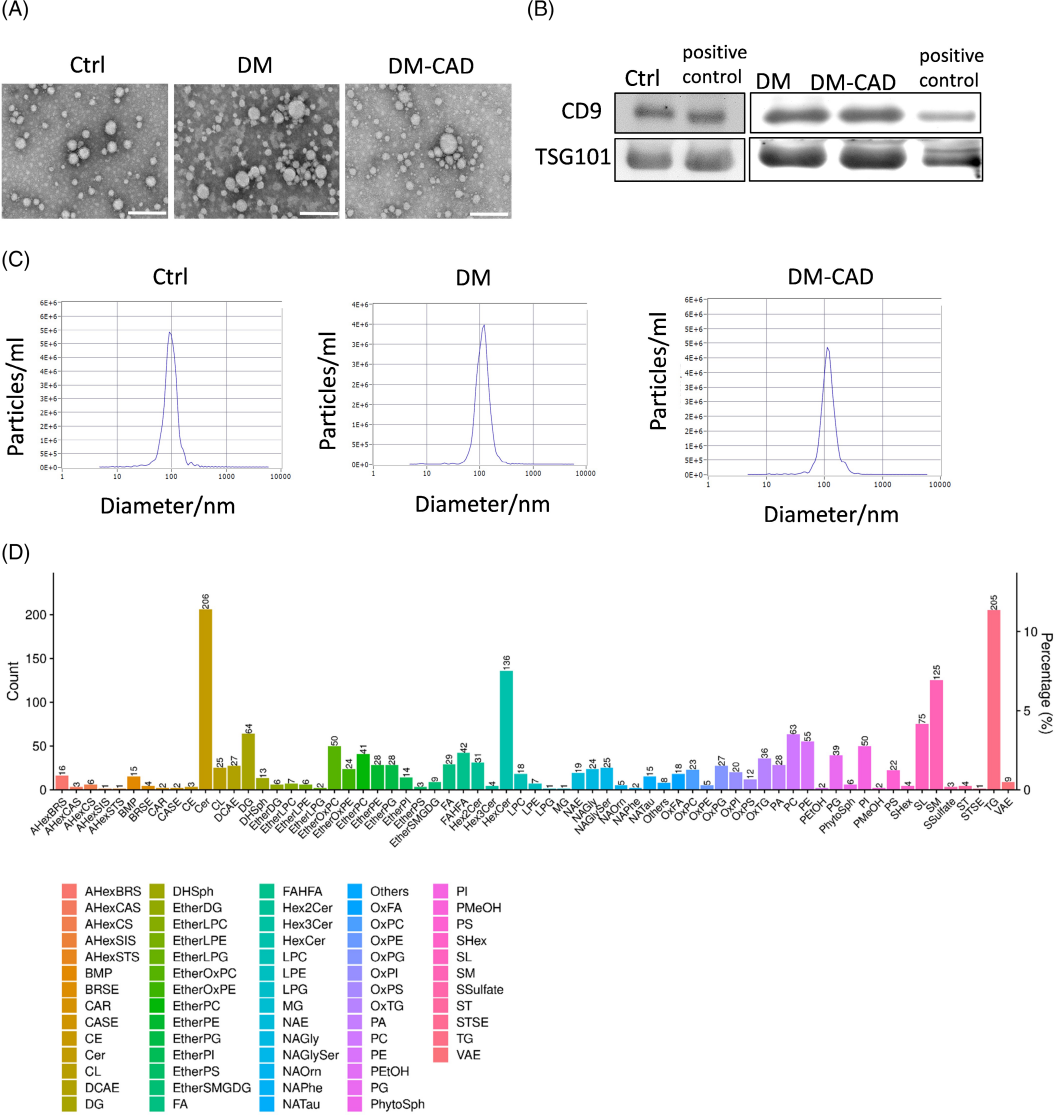
Isolation and characterization of small extracellular vesicles (sEVs) from serum of diabetic patients with or without cardiac dysfunction. (A) sEVs were isolated from serum of control, DM, and DM‐CAD patients by commercial kits with 6 independent replicates. Electron microscopy images of sEVs isolated from serum of control, DM, and DM‐CAD. (B) CD9 and TSG101 of sEVs from different groups were detected by western blots. Scale bar: 100 nm. (C) Particle distributions of sEVs by nanosight particle tracking system. (D) Lipid classes and relative abundance detected in lipidomic analysis. DM, diabetic patients without cardiovascular diseases; DM‐CAD, diabetic patients with cardiovascular diseases.

We performed a lipidomic analysis of sEVs of Ctrl, DM, and DCM. SEVs were collected from the serum of patients separately by sEVs extraction kit as described in the methods. As shown by the electronic microscope, isolated sEVs of all groups have double membrane structure (Figure 1A), and western blot indicates the sEVs are CD9, TSG101 positive (Figure 1B), indicating some of the sEVs are of MVB origin. In addition, they are distributed in particle diameter mostly around 100 nm (Figure 1C).

### 3.2 UHPLC‐MS/MS‐based lipidomic analysis in sEVs derived from the serum of patients with diabetic cardiomyopathy

Lipidomic analysis of sEVs of three groups has been performed. A total of 69 lipid classes are identified in sEVs in all three groups, including 2069 lipid species. Of all the lipid classes, we analyzed high abundance glycerides, including(PC), PE, phosphatidylserine (PS), PG, SM, and triacylglycerol (TG), and diacylglycerol (DG) (Figure 1D).

**FIGURE 2:**
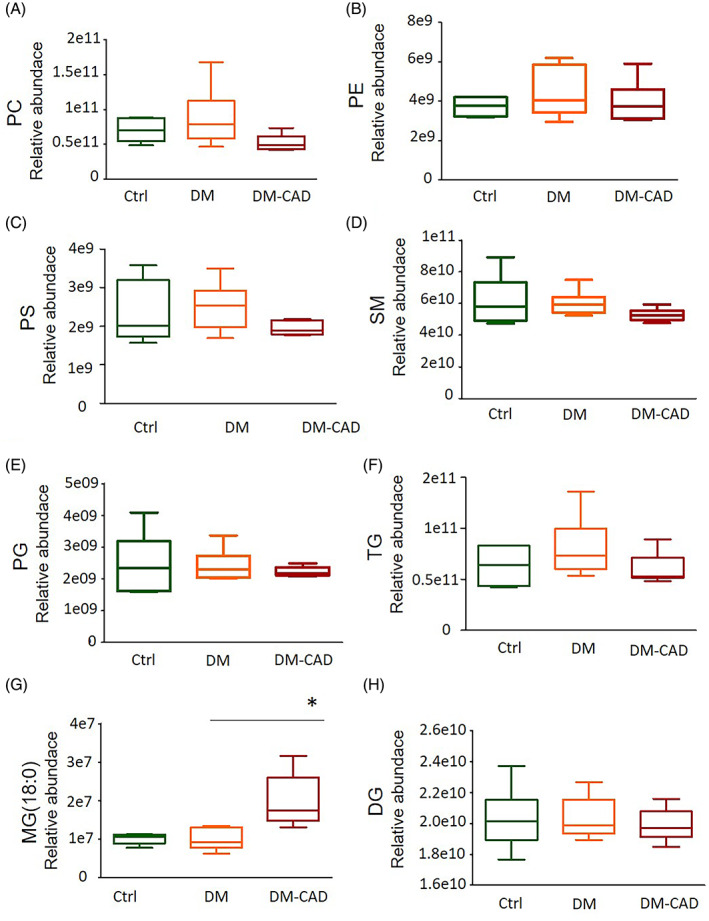
Relative abundance of glycerides by lipidomic analysis. (A) Phosphatidylcholine (PC); (B) Phosphatidylethanolamine (PE); (C) Phosphatidylserine (PS); (D) Sphingomyelin (SM); (E) Phosphatidylglycerol (PG); (F) Triacylglycerol (TG); (G) Monoacylglycerol (MG); (H) Diacylglycerol (DG). The bar graphs indicate the mean ± SEM of six independent replicates. #*p* < .05. DM, diabetic patients without cardiovascular diseases; DM‐CAD, diabetic patients with cardiovascular diseases.

**FIGURE 3:**
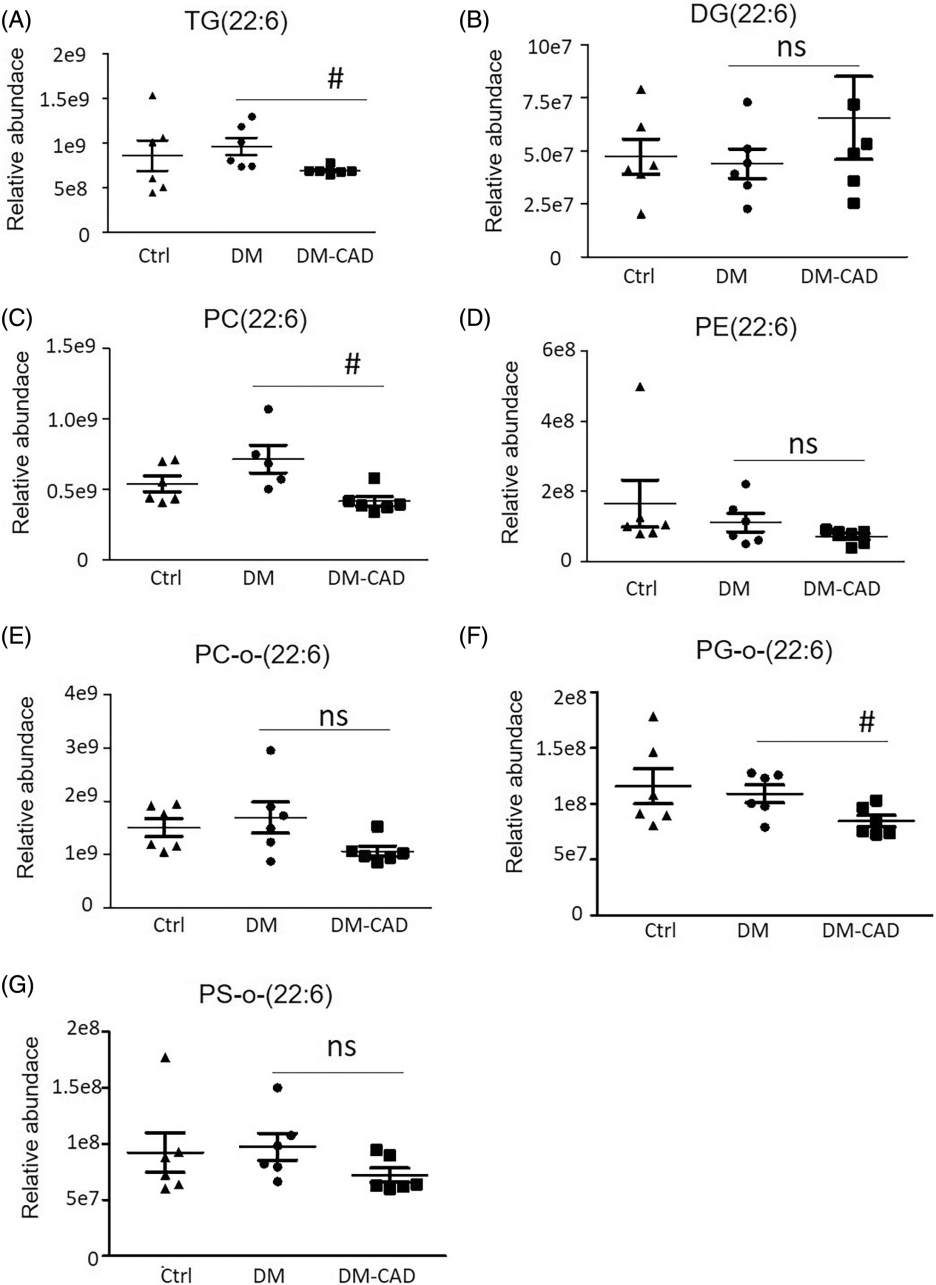
Relative abundance of n‐3 DHA (22:6) enriched glycerides by lipidomic analysis. Relative abundance of (A) TG(22:6); (B) DG(22:6); (C) PC(22:6); (D) PE(22:6); (E) PC‐O‐(22:6); (F) PG‐O‐(22:6); (G) PS‐O‐(22:6). The bar graphs indicate the mean ± SEM of 6 independent replicates. #*p* < .05. DG, diacylglycerol; DHA, docosahexaenoic acid; DM, diabetic patients without cardiovascular diseases; DM‐CAD, diabetic patients with cardiovascular diseases; NS, no significant difference; PC, phosphatidylcholine; PE, phosphatidylethanolamine; PG, phosphatidylglycerol; PS, phosphatidylserine; TG, triacylglycerol.

The results showed no significance in all these high‐abundance lipid classes in all three groups (Figure 2A–F). As reports showed that n‐3 unsaturated fatty acid (PUFA) bond to glycerides is important for biological functions,^17^, ^18^ we further analyze whether there are changes in lipid species with n‐3 PUFA. Surprisingly, the specific lipid classes, docosahexaenoic acid (also known as DHA, 22:6) enriched lipids, have shown different changes, as shown in Figure 3, DHA‐enriched TG and PC are significantly decreased in the DM‐CAD group compared to the DM group (Figure 3A), but this is not observed in DHA‐enriched PE and DG. Moreover, oxidized PC (PC‐O) and PG (PG‐O) also showed a decrease in DHA‐enriched species (Figure 3E,F), but not for oxidized PS (PS‐O) (Figure 3G).

**FIGURE 4:**
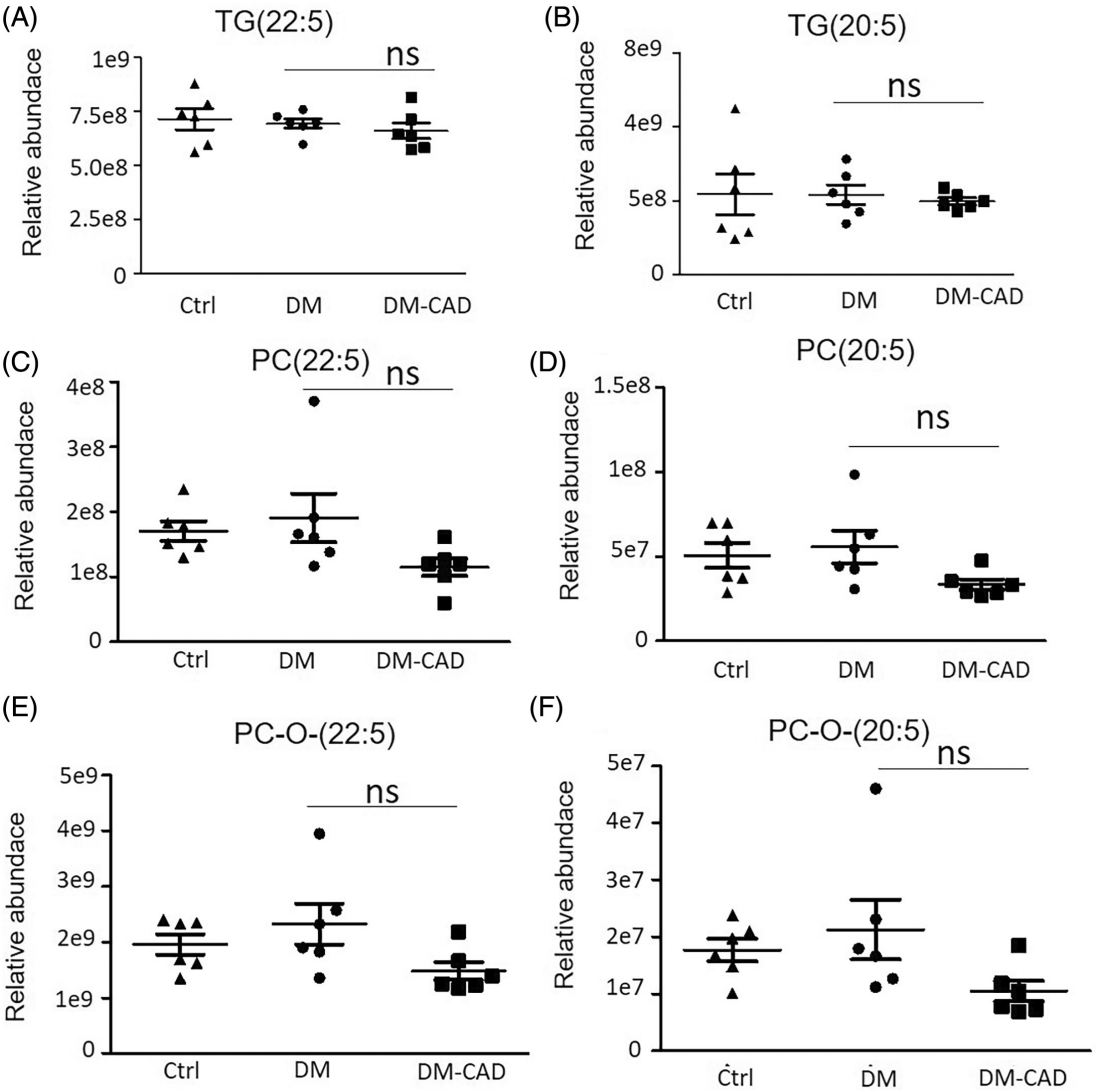
Relative abundance of n‐3 EPA (20:5) and DPA (22:5) enriched glycerides by lipidomic analysis. Relative abundance of (A)TG(22:5); (B) TG(20:5); (C) PC(22:5) ; (D) PC(20:5); (E) PC‐O‐(22:5); (F) PC‐O‐(20:5). The bar graphs indicate the mean ± SEM of 6 independent replicates. #*p* < .05. DM, diabetic patients without cardiovascular diseases; DM‐CAD, diabetic patients with cardiovascular diseases; DPA, docosapentaenoic acid; EPA, eicosapentaenoic acid; PC, phosphatidylcholine; TG, triacylglycerol.

As shown in the figures, TG, PC, and PC‐O has relatively high abundance in sEVs, we further analyze whether other essential fatty acid‐enriched TG, PC, and PC‐O species are changed, as shown in Figure 4, docosapentaenoic acid‐22:5 (DPA) or eicosapentaenoic acid‐20:5 (EPA)‐enriched TG has no significant changes in all three groups (Figure 3B,C). DPA‐ or EPA‐enriched PG and PG‐O has a tendency to decrease in the DM‐CAD group but has no significant difference.

### 3.3 sEVs diminished the insulin signaling pathway of AC16 cells

**FIGURE 5:**
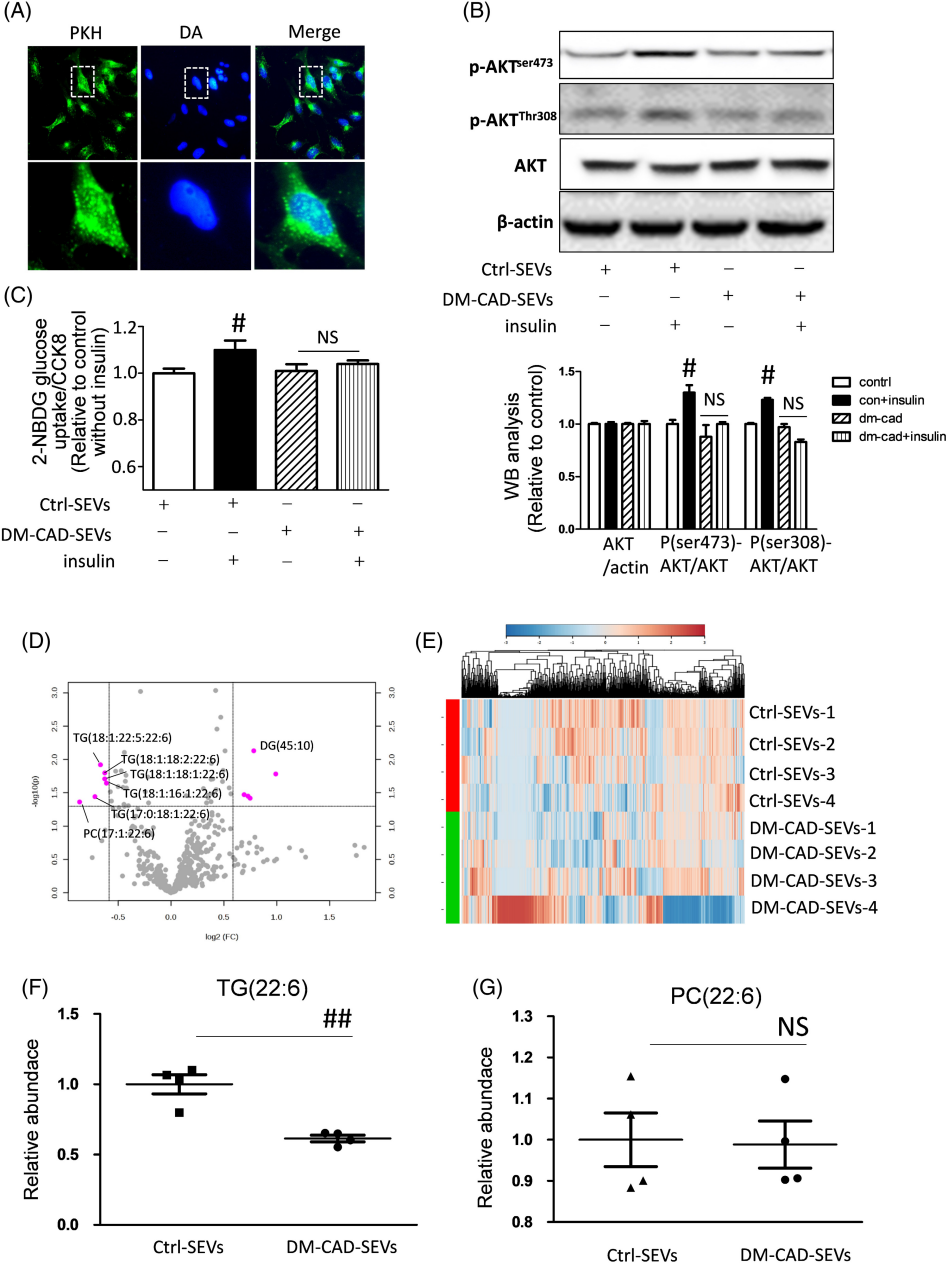
Decreased of n‐3 DHA enriched glycerides by serum sEVs from DM‐CAD group in AC16 cells. (A) sEVs can be uptake by AC16 cells; (B) sEVs derived from DM‐CAD group diminished phosphorylation of AKT at Ser473 and Thr308 site; (C) Insulin stimulated 2‐NBDG glucose uptake were decreased by sEVs‐DM‐CAD treatment; (D) Volcano blot of fold changes of individual lipid molecules in AC16 cells treated with control‐sEVs or DM‐CAD‐sEVs; (E) A heat map of lipidomic analysis in AC16 cells; (F, G) Relative abundance of n‐3 DHA (22:6) enriched TG and PC in AC16 cells. #*p* < .05. DHA, docosahexaenoic acid; DM, diabetic patients without cardiovascular diseases; DM‐CAD, diabetic patients with cardiovascular diseases; NS, no significant difference; PC, phosphatidylcholine; sEV, small extracellular vesicle; TG, triacylglycerol.

In the following study, we examined if the plasma sEVs can enter into cardiomyocytes, first, we labeled the sEVs with PKH67, which is a membrane fluorescence agent, and added the labeled sEVs into culture media of human origin cell line AC16, the results showed that sEVs from plasma can enter into cardiac cells (Figure 5A), and sEVs from DM‐CAD patients can diminish insulin sensitivity in normal AC16 cells, as indicated in Figure 5B, both ser473 and thr308 phosphorylation of AKT response to insulin stimuli are decreased by sEVs‐DM‐CAD treatment, but not by sEVs‐control treatment. In addition, 2‐NBDG glucose uptake assay showed glucose uptake stimulated by insulin is decreased by sEVs‐DM‐CAD treatment but not sEVs‐control treatment (Figure 5C).

### 3.4 Comparison of lipid components between sEVs and sEVs treated AC16 cells

To further understand the role of sEVs‐DM‐CAD on cells, we perform lipidomics assays on cells treated with sEVs from the control group and the DM‐CAD group. We compared this result with previous lipidomic analysis of plasma sEVs and found that TG (18:1, 22:5, 22:6), TG (18:1, 18:2, 22:6), TG (18:1, 16:1, 22:6), TG (17:0, 18:1, 22:6) were decreased compared to control sEVs treatment group also PC (17:1, 22:6) docosahexaenoic acid decreased compared to control (Figure 5D,E). Additionally, the relative abundance of TG species with DHA are decreased in the DM‐CAD treated group compared to the control group (Figure 5F), but the PC species with 22:6 have no significant changes (Figure 5G).

## 4 DISCUSSION

In recent years, the ability of sEVs to mediate cell–cell communication and crosstalk between organs has attracted great interest in exploring the biological function of sEVs. In diabetic conditions, sEVs derived from different tissues affected cellular functions in different ways. Experimental study shows that sEVs from bone marrow stem cells increase the gene expression of insulin signal pathway (insulin, Pdx1, Smad2, Smad3, and transforming growth factor β) in the type 1 diabetes condition and alleviate insulin resistance in the T2D rat model.^19^, ^20^ In addition, sEVs derived from adipose‐derived stem cells have the potential to be used in the clinic to promote wound healing in patients with diabetes.^21^ Circulating sEVs are mostly derived from hemopoietic cells.^22^ Cargoes carried by circulating sEVs are found to be modulators or biomarkers for diabetes and its complications, for example, miR‐122‐5p shuttling by EVs regulates the viability and apoptosis of cardiomyocytes in diabetic rodent models.^23^ Arginase1 carried by serum sEVs induces endothelial dysfunction in diabetes.^12^ In addition, some insulin‐signaling proteins, such as p70S6K, p‐GSK3β, p‐Akt, and p‐insulin receptor, are contained in serum sEVs, indicating its association with incidences of insulin resistance.^24^

Lipid profiles are an important mechanism for maintaining cellular insulin activity. Lipids of lower carbon number and double bond content were associated with an increased risk of diabetes, whereas lipids of higher carbon number and double bond content were associated with decreased risk. This pattern was strongest for TG and persisted after multivariable adjustment for age, sex, BMI, fasting glucose, fasting insulin, total triglycerides, and high‐density lipoprotein (HDL) cholesterol.^25^ EPA (20:5) and DHA (22:6) are well‐known n‐3 PUFAs that are normally ingested by consumption of fish oils. EPA and DHA were shown to have health benefits in cardiovascular diseases by reprogramming triglyceride‐rich lipoproteins metabolism, reducing inflammatory mediators (cytokines and leukotrienes), and modulating cell adhesion molecules.^26^ Moreover, evidence suggests that DHA is more efficient in decreasing blood pressure, heart rate, and platelet aggregation compared to EPA.^27^ Recent studies suggest that n‐3 PUFA in fish oil is primarily esterified as TG,^28^ which was shown to have higher bioavailability and lower oxidation rate than free style. n‐3 PUFA‐TG in the diet significantly decreased the serum total cholesterol and non‐HDL cholesterol.^29^ In diabetic condition, dysfunction of lipid TGs are associated with the incidence of diabetes. TGs can be carried by sEVs and promote or protect against diabetes and its complications directly or indirectly. In particular, TG (12:0_18:2_22:6), TG (16:0_11:1), TG (49:0), (TG 51:1), and DG (18:2_22:6) were independently associated with increased T2D risk.^30^ In our study, we found that total TG and PC content has no significant difference between the control, DM, and DM‐CAD groups. DHA‐enriched TG and PC are significantly decreased in the DM‐CAD group compared to the DM group. More important, after being treated with DM‐CAD‐sEVs in AC16 cells, DHA‐enriched TG, including TG (18:1, 18:2, 22:6), TG (18:1, 18:1, 22:6), TG (18:1, 16:1, 22:6), TG (18:1, 22:5, 22:6), and TG (17:0, 18:1, 22:6), are also decreased compared to the control group, suggesting the component of TGs in sEVs constitute an important part of DHA‐TG in AC16 cells.

## 5 LIMITATIONS AND CONCLUSIONS

We performed a widely targeted quantitative lipidomic analysis of serum sEVs of healthy controls and diabetic patients with or without cardiovascular disease. In addition, we performed a lipidomic analysis of AC16 cells that were treated with sEVs derived from DM‐CAD patients and revealed differential lipid compositions between groups. Comparing sEVs from DM and DM‐CAD, sEVs from DM‐CAD lacked with DHA‐TG species. We further demonstrated the sEVs from DM‐CAD could diminish insulin‐stimulated phosphorylation of AKT in AC16 cells. However, due to the limitation of relative quantitative nature  in nontargeted lipodomics,   and lipid classes detection by nontargeted lipodomics are not comprehensive, a targeted lipidomic analysis on TG species should be performed. Also, these potential biomarkers need to be validated in a larger cohort. Additionally, further in vitro experimental studies should be conducted to confirm the biological function of DHA‐TG in cell signal transduction. Overall, our findings make explicit the concept that the DHA‐TG component of sEVs from serums may mediate intercellular signaling and be associated with the incidence of diabetic cardiovascular complications.

## AUTHOR CONTRIBUTIONS

Wei Ding, Xuejuan Zhang, and Dandan Xiao collected samples and performed the experiments. Wenguang Chang designed the experiment and wrote the manuscript. All authors have read and approved the final version of the manuscript.

## FUNDING INFORMATION

This work was supported by Natural Science Foundation of Shandong Province (ZR2019ZD28).

## CONFLICT OF INTEREST STATEMENT

The authors confirm that there are no conflicts of interest.
